# Presynaptic NMDA Receptors Influence Ca^2+^ Dynamics by Interacting with Voltage-Dependent Calcium Channels during the Induction of Long-Term Depression

**DOI:** 10.1155/2022/2900875

**Published:** 2022-02-07

**Authors:** Florian B. Neubauer, Rogier Min, Thomas Nevian

**Affiliations:** ^1^Department of Physiology, University of Bern, Bühlplatz 5, 3012 Bern, Switzerland; ^2^Department of Integrative Neurophysiology, Center for Neurogenomics and Cognitive Research, Vrije Universiteit Amsterdam, Netherlands; ^3^Department of Child Neurology, Emma Children's Hospital, Amsterdam University Medical Centers, Amsterdam Neuroscience, Vrije Universiteit Amsterdam, Netherlands

## Abstract

Spike-timing-dependent long-term depression (t-LTD) of glutamatergic layer (L)4-L2/3 synapses in developing neocortex requires activation of astrocytes by endocannabinoids (eCBs), which release glutamate onto presynaptic NMDA receptors (preNMDARs). The exact function of preNMDARs in this context is still elusive and strongly debated. To elucidate their function, we show that bath application of the eCB 2-arachidonylglycerol (2-AG) induces a preNMDAR-dependent form of chemically induced LTD (eCB-LTD) in L2/3 pyramidal neurons in the juvenile somatosensory cortex of rats. Presynaptic Ca^2+^ imaging from L4 spiny stellate axons revealed that action potential (AP) evoked Ca^2+^ transients show a preNMDAR-dependent broadening during eCB-LTD induction. However, blockade of voltage-dependent Ca^2+^ channels (VDCCs) did not uncover direct preNMDAR-mediated Ca^2+^ transients in the axon. This suggests that astrocyte-mediated glutamate release onto preNMDARs does not result in a direct Ca^2+^ influx, but that it instead leads to an indirect interaction with presynaptic VDCCs, boosting axonal Ca^2+^ influx. These results reveal one of the main remaining missing pieces in the signaling cascade of t-LTD at developing cortical synapses.

## 1. Introduction

Presynaptic NMDA receptors (preNMDARs) have important functions in synaptic transmission, information processing, and long-term plasticity in several regions of the brain [1–3]. Particularly, preNMDARs are thought to be required for the induction of spike-timing-dependent LTD (t-LTD) at developing neocortical synapses [4–7]. Recently, we suggested that t-LTD in the developing rat barrel cortex requires eCB-dependent activation of astrocytes, which results in the release of glutamate onto preNMDARs [8, 9]. Importantly, we showed that astrocyte activity alone is not sufficient for the induction of LTD. Simultaneous presynaptic APs concomitant with astrocyte activation are required [8]. This suggests that axonal APs interact with preNMDARs in a yet unknown way leading to the induction of t-LTD.

Under certain conditions, preNMDARs in barrel cortex can also function as autoreceptors for presynaptically released glutamate. Bursts of APs followed by a correctly timed additional AP can induce pattern-dependent LTD at the L4-L2/3 synapse [10]. Importantly, since this form of LTD requires presynaptic release of glutamate, it bypasses the need for astrocyte activation. Similarly, bursts of APs in the visual cortex can induce preNMDAR dependent LTD at L4-L4 connections [11]. Therefore, preNMDAR-mediated LTD always requires presynaptic activity, whereas the source of the glutamate (astrocytic or presynaptic) might vary. This implies that there should be a presynaptic coincidence detection mechanism involving both presynaptic activity and preNMDAR activation.

Despite the studies highlighted above, there is an active debate about the existence and function of preNMDARs. This debate is mainly fueled by contradictory findings on presynaptic Ca^2+^ signals mediated by preNMDARs. Lack of axonal Ca^2+^ signals in boutons of L5 and L4 neurons in developing neocortex upon iontophoresis of aspartate or upon MNI-glutamate uncaging, and lack of an APV sensitive component in single AP-evoked Ca^2+^ transients argue against the existence of preNMDARs [12–14]. On the other hand, there is ample anatomical and physiological evidence for the presence of preNMDARs [1, 2]. Yet, their mechanism of function remains elusive. It has been hypothesized that either a direct presynaptic Ca^2+^ influx through preNMDARs, a presynaptic depolarization mediated by Na^+^ influx through preNMDARs [15] or a metabotropic effect [16], could be the mechanism of preNMDAR function. The properties of preNMDARs strongly depend on their subunit composition, which can influence permeability for Ca^2+^, voltage-sensitive Mg^2+^ block, subcellular location, and gating kinetics. preNMDARs at cortical synapses contain GluN2C, GluN2D, or GluN3A subunits, rendering them relatively Mg^2+^ insensitive with low permeability for Ca^2+^ [17, 18]. Consistently, a direct presynaptic Ca^2+^ influx through preNMDARs at cortical synapses has rarely been observed. At a fraction of cortical L5 boutons, pairing activation of preNMDARs by glutamate uncaging and high-frequency AP firing causes an enhancement of axonal Ca^2+^ influx [19]. In contrast, preNMDARs located on parallel fibres in the cerebellum seem to be permeable for Ca^2+^, and a direct preNMDAR-mediated Ca^2+^ influx has been reported [20].

Here, we hypothesize that the activation of preNMDARs alone is not sufficient to evoke a detectable Ca^2+^ signal in L4 boutons in developing barrel cortex, but that the interaction with axonal APs is required. To investigate this hypothesis, we utilized a form of chemical LTD mediated by bath application of 2-AG combined with presynaptic Ca^2+^ imaging. First, we showed that eCB-LTD depended on astrocyte and preNMDAR activation. Bath application of 2-AG should globally activate astrocytes that innervate preNMDARs, thus, increasing the probability to detect an influence on presynaptic Ca^2+^ dynamics. Indeed, we observed an APV-sensitive broadening of AP-evoked Ca^2+^ transients. Investigation of the underlying mechanism suggested that preNMDARs have little Ca^2+^ permeability and that their major mechanism of function is to interact with voltage-dependent Ca^2+^ channels (VDCCs) to prolong AP-evoked Ca^2+^ influx in presynaptic boutons. Our data is consistent with previous results and can help to reconcile apparent contradictory observations, thereby contributing to a better mechanistic understanding of preNMDAR function.

## 2. Material and Methods

### 2.1. Slice Preparation

Experiments were approved by the Veterinary Office of the Canton of Bern, Switzerland. Thalamocortical brain slices containing the barrel subfield of somatosensory cortex were prepared from 12–21 d old Wistar rats of either sex [21]. Rats were decapitated, and their brains were quickly removed into cold (0–4°C) oxygenated physiological solution containing 125 mM NaCl, 2.5 mM KCl, 1.25 mM NaH_2_PO_4_, 25 mM NaHCO_3_, 1 mM MgCl_2_, 2 mM CaCl_2_, and 25 mM glucose. Slices, 300 *μ*m thick, were cut from the tissue block with a vibratome (Microm) and kept at 37°C for 30 min and then at room temperature until use.

### 2.2. Electrophysiology

All experiments were performed at 30–34°C. For recording, slices were transferred to a recording chamber perfused with oxygenated physiological solution (same as above). The barrel subfield of somatosensory cortex was identified by the presence of barrels in L4, visible under trans-illumination. A monopolar glass stimulation electrode was placed in a L4 barrel, and whole-cell recordings for plasticity experiments were performed from L2/3 pyramidal neurons right above the corresponding barrel. Cells were identified using infrared gradient contrast video microscopy. Recording electrodes with a resistance of 4–7 M*Ω* were made using borosilicate glass capillaries. Recordings were performed using Dagan BVC-700A amplifiers (Dagan). Data were acquired with an ITC-16 AD-DA board (Instrutech) and using Igor software (Wavemetrics). The intracellular solution for recording neurons contained 130 mM potassium gluconate, 10 mM potassium HEPES, 10 mM sodium phosphocreatine, 4 mM Mg-ATP, 0.3 mM Na-GTP, 4 mM NaCl, 10 mM sodium gluconate (pH 7.3 with KOH), and biocytin (0.2% w/v).

Single component EPSPs in the pyramidal neuron with amplitudes between 1 and 5 mV were evoked by stimulation in L4. After obtaining a stable baseline for 10 min at 0.1 Hz stimulation, the endocannabinoid 2-AG (10-20 *μ*M) was bath applied for 20 min, while continuing the extracellular stimulation. During wash-out, EPSPs were recorded for an additional 40 min. Experiments were discarded if the baseline EPSP slope was unstable (>10% change between first 15 and last 15 EPSP slopes of the baseline period), or if the pyramidal neuron input resistance or membrane potential changed by >15% during the course of the experiment.

To investigate the influence of astrocytes on synaptic depression, whole-cell patch clamp recordings were performed from astrocytes adjacent to the recorded pyramidal neuron in L2/3. The intracellular solution for recording astrocytes contained 135 mM KCH_3_O_3_S, 10 mM HEPES, 10 mM sodium phosphocreatine, 4 mM MgCl_2_, 4 mM Na_2_-ATP, and 0.4 mM Na-GTP (pH 7.2 with KOH). Astrocytes were characterized by a low resting membrane potential, passive responses to both negative and positive current injections, and a low membrane resistance [8]. For astrocyte Ca^2+^ clamp experiments, 200 *μ*M OGB-1, 0.45 mM EGTA, and 0.14 mM CaCl_2_ were added to the astrocyte intracellular solution to clamp intracellular free Ca^2+^ at a steady-state concentration of 50–80 nM [22].

For axonal Ca^2+^ imaging experiments, recordings from spiny stellate neurons in L4 of the barrel cortex were performed in the whole-cell current clamp configuration. The intracellular solution was supplemented with the Ca^2+^ indicator Oregon Green Bapta-1 (OGB-1, 200 *μ*M) and the morphological dye Alexa-594 (50 *μ*M). APs were evoked by suprathreshold somatic current injections (5 ms) at varying frequencies.

Ionotophoresis of glutamate (100 *μ*M) through a high resistance (>100 M*Ω*) application glass pipette was performed with an AxoClamp 2B amplifier in current clamp mode. A small retain current was applied to prevent leakage of glutamate. Brief (1 ms) current pulses were used to iontophorese the glutamate in close proximity to dendrites or axons of L4 spiny stellate neurons.

### 2.3. Ca^2+^ Imaging

For two-photon excitation fluorescence microscopy, an infrared femtosecond-pulsed titanium sapphire laser (MaiTai, Spectraphysics) was coupled to a home-built laser scanning microscope equipped with a water-immersion objective (W63x HCX APO UVI, 0.9 NA, Leica). Excitation infrared laser light and fluorescence emission light were separated at 670 nm (excitation filter 670DCXXR, AHF Analysentechnik). The emission spectra were separated by a dichroic mirror at 560 nm (beam splitter 560DCXR, AHF) and corresponding bandpass (HQ525/50, HQ610/75, AHF) and infrared-block filters (700SP-2P, AHF) and were detected using nondescanned detection behind the objective. Dyes were excited at *λ* = 920 nm. Data was acquired using custom-written laser-scanning software in LabView (National Instruments) [23]. Axonal Ca^2+^ imaging was performed in frame scan mode (30 × 30 *μ*m^2^) at 3 Hz for 1 min duration and repeated every 5 min. Astrocytic Ca^2+^ signals were acquired from astrocytes loaded with Rhod2-AM by pressure ejection of the dye-containing solution into the brain slice under visual control with a 10× objective. For dye preparation, 50 *μ*g Rhod2-AM was dissolved in 5 *μ*l of 80% DMSO and 20% pluronic acid F127 (*w*/*v*; Sigma) and diluted 1 : 19 in a HEPES-buffered solution containing 125 mM NaCl, 2.5 mM KCl, and 10 mM HEPES. This procedure resulted in specific uptake of the Rhod2 in astrocytes in the injected area. Frame scans (35 × 35 *μ*m^2^) at 3 Hz for 2 min duration, repeated every 5 min containing one astrocyte, were performed before, during, and after bath application of 2-AG.

### 2.4. Data Analysis

Electrophysiological data were analyzed using custom-written procedures in Igor Pro (Wavemetrics). EPSP slope was measured as a linear fit between time points on the rising phase of the EPSP corresponding to 20 and 60% of the EPSP peak amplitude. The change in EPSP slope was evaluated 20–40 min after the end of the pairing period and normalized to the baseline EPSP slope. Axonal and astrocyte imaging data were analyzed using custom-written procedures in Matlab. Regions of interest containing the axon segment were automatically detected and fluorescence traces extracted. Relative fluorescence changes were calculated as Δ*F*/*F* = (*F*(*t*) − *F*_0_)/*F*_0_, where *F*(*t*) denotes fluorescence over time and *F*_0_ baseline fluorescence. AP-evoked Ca^2+^ transients were normalized and averaged. Single exponential fits to the decay of the Ca^2+^ transients yielded the decay time constants for the different conditions.

### 2.5. Statistical Analysis

Statistical analysis was done using paired or unpaired Student's *t*-test (for single comparisons) or ANOVA with post hoc Bonferroni correction (for multiple comparisons to the same control). Statistical significance was asserted for *p* < 0.05. Data are presented as mean ± s.e.m.

### 2.6. Histology

During experiments, cells were filled with biocytin and fixed in 4% paraformaldehyde. Slices were developed with the avidin-biotin-peroxidase method and mounted on cover slides for reconstruction with Neurolucida [24, 25].

### 2.7. Chemicals

Chemicals were obtained from the following sources: 2-AG and cyclosporin-A from Sigma-Aldrich, 1-(2,4-dichlorophenyl)-5-(4-iodophenyl)-4-methyl-N-(piperidin-1-yl)-1H-pyrazole-3-carboxamide (AM251), d-AP5 from Ascent Scientific, (+)-5-methyl-10,11-dihydro-^5^H-dibenzo[a,d]cyclohepten-5,10-imine maleate (MK-801), and L-glutamic acid from Tocris, FK506 from Abmole Bioscience.

## 3. Results

### 3.1. Endocannabinoid-Dependent LTD Requires Activation of Astrocytes and preNMDARs

**Figure 1:**
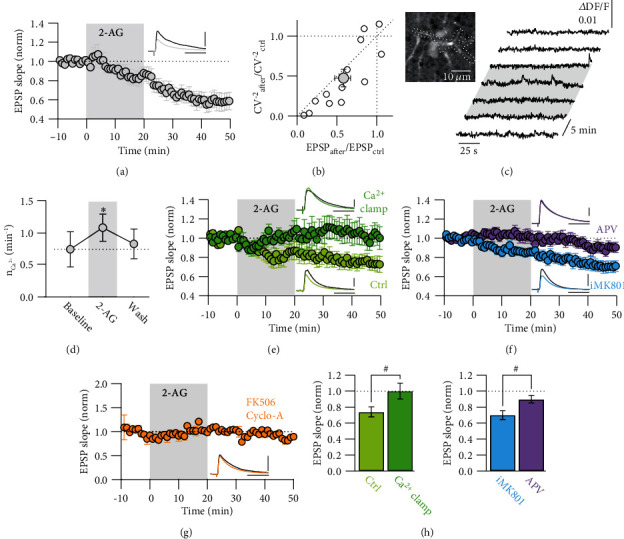
2-AG mediated LTD requires astrocyte Ca^2+^ signaling and preNMDARs. (a) Time course of normalized and averaged EPSP slope measured in L2/3 pyramidal neurons before, during (0–20 min, shaded area) and after bath application of 2-AG (*n* = 13). Inset, representative average EPSP during baseline (black) and after 2-AG (grey). (b) Relative change of coefficient of variation of EPSP slope after bath application of 2-AG as a function of corresponding changes in EPSP slope. The relation is almost linear, indicating a presynaptic locus of eCB-LTD expression. Open circles represent individual experiments, and filled circle represents the average (*n* = 13). (c) Two-photon fluorescence image of an astrocyte in L2/3 of the somatosensory cortex loaded with the Ca^2+^ indicator Rhod-2. Traces to the right show Ca^2+^ fluctuations in the astrocyte before, during, and after bath application of 2-AG (shaded area). (d) Summary of the average number of Ca^2+^ transients during the time course of the experiment (*n* = 12). ^∗^*p* < 0.05 for the effect of time on Ca^2+^ transient number by Student's paired *t*-test. (e) Normalized and averaged EPSP slope over time in L2/3 pyramidal neurons, while an adjacent astrocyte was infused in the whole-cell recording configuration with either a control (Ctrl, *n* = 8) and or Ca^2+^ clamp solution (*n* = 9). Inset, representative average EPSP during baseline (black) and after 2-AG in control (light green) or Ca^2+^ clamp (dark green) conditions. (f) Normalized and averaged EPSP slope over time during bath application of APV (*n* = 13) or intracellular infusion of MK801 (*n* = 24) into the pyramidal neuron (iMK801). Inset, representative average EPSP during baseline (black) and after 2-AG in the presence of APV (purple) or iMK801 (blue). (g) Normalized and averaged EPSP slope in the presence of the calcineurin inhibitors FK506 and cyclosporin-A (*n* = 2). Inset, representative average EPSP during baseline (black) and after 2-AG (orange). (h) Bar graph summary of experiments shown in (e) and (f). In the astrocyte Ca^2+^ clamp condition, eCB-LTD was abolished. APV blocked eCB-LTD, while intracellular block of postsynaptic NMDARs with MK801 had no effect. ^#^*p* < 0.05 by one-way ANOVA. All data are represented as mean ± SEM. All scale bars for average EPSPs represent 40 ms and 2 mV, respectively.

t-LTD at L4-L2/3 excitatory synapses in rat barrel cortex depends on the activation of astrocytes by 2-AG that is synthetized postsynaptically. This synthesis occurs at synapses which are activated within a time window of about 50 ms after the generation of a postsynaptic AP. Astrocyte activation results in the release of glutamate onto preNMDARs which interact with presynaptic APs to trigger a reduction in release probability [8]. To further understand the presynaptic signaling cascade leading to t-LTD, Ca^2+^ imaging from presynaptic boutons during t-LTD induction would be the best approach. However, this is technically challenging, since it requires imaging from the presynaptic bouton of an identified synaptic connection while at the same time controlling AP firing in the pre- and postsynaptic neuron. As an alternative approach, we tested if direct bath-application of 2-AG resulted in synaptic depression without postsynaptic activity at L4-L2/3 synapses, similar to what was previously described for L5-L5 synapses [4]. We performed whole-cell patch-clamp recordings from L2/3 pyramidal neurons in the somatosensory cortex of juvenile rats and activated L4 spiny stellate neuron axons by extracellular stimulation in L4. After recording a baseline of EPSPs for 10 min, we bath-applied 2-AG (10 *μ*M) for 20 min while continuing presynaptic stimulation at 0.1 Hz. We observed a long-lasting depression of the EPSPs 10–30 min after the washout of 2-AG (0.58 ± 0.09, *n* = 13, *p* < 0.01 for the effect of time on EPSP slope by Student's paired *t*-test; Figure 1(a)). This form of LTD was presynaptic as the reduction in normalized EPSP amplitude correlated with a reduction in the normalized coefficient of variation (Figure 1(b)).

Next, we confirmed that 2-AG activated cortical astrocytes in L2/3. Bulk-loading of astrocytes with Rhod2-AM, which is preferentially taken up by astrocytes, allowed to measure the intracellular Ca^2+^ dynamics during bath-application of 2-AG (Figure 1(c)). We observed a significant increase in the number of Ca^2+^ transients by 2-AG (from 0.75 ± 0.26 min^−1^ to 1.08 ± 0.20 min^−1^, *n* = 12, *p* < 0.05 for the effect of time on Ca^2+^ transient number by Student's paired *t*-test) that decayed back to baseline levels after wash-out (Figure 1(d)). This experiment confirmed previous results showing that eCBs modulate astrocytic Ca^2+^ dynamics [8, 26]. Infusing an astrocyte with a solution that clamped the intracellular Ca^2+^ concentration to a constant level abolished eCB-LTD in adjacent pyramidal neurons (1.00 ± 0.10, *n* = 9, *p* = 0.87 for the effect of time on EPSP slope by Student's paired *t*-test), while infusing a control intracellular solution into the astrocytes resulted in eCB-LTD (0.74 ± 0.06, *n* = 8, *p* < 0.05 for the effect of time on EPSP slope by Student's paired *t*-test; comparison control vs. Ca^2+^ clamp, *p* < 0.05 by one-way ANOVA; Figures 1(e) and 1(h)). Thus, similar to t-LTD, the increase in Ca^2+^ signaling in the astrocytes was required for the induction of eCB-LTD.

Then, we tested the involvement of NMDARs in eCB-LTD. Bath-application of APV blocked the 2-AG mediated LTD (0.90 ± 0.05, *n* = 13, *p* = 0.06 for the effect of time on EPSP slope by Student's paired *t*-test), while infusion of MK801 into the postsynaptic cell had no effect on eCB-LTD (0.70 ± 0.05, *n* = 24, *p* < 0.001 for the effect of time on EPSP slope by Student's paired *t*-test; comparison APV vs. MK801, *p* < 0.05 by one-way ANOVA; Figures 1(f) and 1(h)). These results suggested that downstream of astrocyte signaling, eCB-LTD required the activation of presynaptic NMDARs.

PreNMDAR-dependent LTD at cortical synapses [10] and eCB-mediated LTD at both excitatory and inhibitory synapses in the hippocampus [27, 28] require the activation of the Ca^2+^ dependent protein phosphatase calcineurin [29]. In order to test a similar involvement in our case, we blocked calcineurin activity by incubating the brain slices in FK506 (50 *μ*M) and cyclosporin-A (25 *μ*M) at least for 1 h before the start of the experiment and with 20 *μ*M and 10 *μ*M, respectively, during the experiment. EPSPs were evoked by extracellular stimulation in L4, and 2-AG was washed-in for 20 min as described above. In the condition of blocked calcineurin activity, no eCB-LTD was induced (0.95 ± 0.01, *n* = 2, Figure 1(g)). These results indicate that calcineurin is involved in the induction of eCB-LTD.

In summary, our experiments show that eCB-LTD and t-LTD share a similar induction mechanism, since both are dependent on astrocyte activation by eCBs and on activation of preNMDARs. The involvement of calcineurin suggests that an elevation in presynaptic Ca^2+^ is essential for these forms of LTD.

### 3.2. Presynaptic NMDARs Broaden AP-Evoked Ca^2+^ Transients in L4 Spiny Stellate Axons

**Figure 2:**
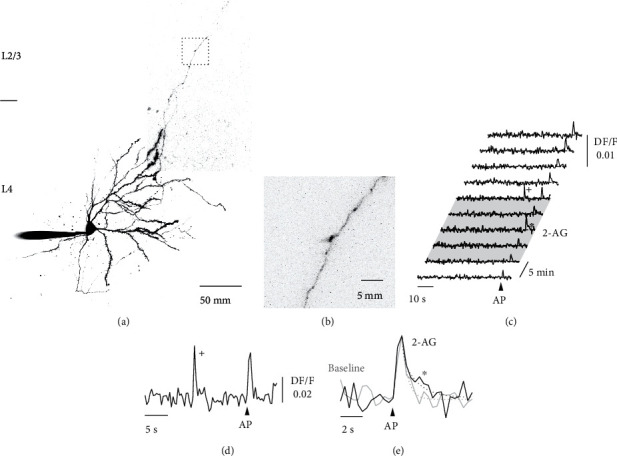
Presynaptic Ca^2+^ imaging in L4 spiny stellate axons. (a) Two-photon fluorescence image of a spiny stellate neuron in L4 of the somatosensory cortex loaded with OGB-1 and Alexa-594. (b) Imaged axon segment in L2/3 indicated in (a) by the dashed box. (c) Consecutive fluorescence traces of 1 min duration repeated every 5 min before, during (shaded area) and after bath application of 2-AG. An arrowhead indicates the time point of a somatically evoked AP. (d) Spontaneous axonal Ca^2+^ transient marked by a cross in (c) on an expanded scale. The Ca^2+^ transient was unrelated to somatic activity. (e) AP-evoked Ca^2+^ transient in the presence of 2-AG marked by an asterisk in (c) on an expanded scale (black) compared to an AP-evoked Ca^2+^ transient during baseline (grey). Dashed lines present single exponential fits to the decay of the Ca^2+^ transients.

**Figure 3:**
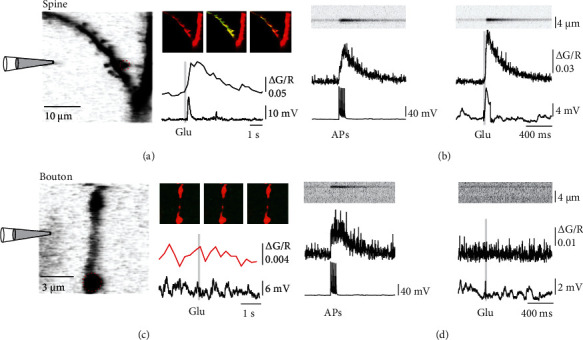
Iontophoresis of glutamate does not evoke Ca^2+^ signals in boutons. (a) Two-photon fluorescence image of a dendrite of a spiny stellate neuron loaded with the Ca^2+^ indicator OGB-1 (200 *μ*M) and the morphological dye Alexa 594 (50 *μ*M). The position of the iontophoresis pipette for glutamate application is indicated. To the right, three fluorescence images taken before, 1 s after and 5 s after glutamate application, are shown. Below, the time-course of the fluorescence change in the region of interest indicated by the red, dashed circle (upper trace) and the somatic membrane potential (lower trace) are presented. Grey bar represents time of glutamate application. (b) Left, line-scan through a spine of another cell and the corresponding Ca^2+^ transient evoked by a burst of 5 APs at 50 Hz. Right, line-scan through the same spine during iontophoresis of glutamate. A clear increase in Ca^2+^ can be seen upon iontophoresis. (c) Two-photon fluorescence image of an axon of a spiny stellate neuron located in L2/3 loaded with the Ca^2+^ indicator OGB-1 (200 *μ*M) and the morphological dye Alexa 594 (50 *μ*M). The position of the iontophoresis pipette for glutamate application is indicated. To the right, three fluorescence images taken before, 1 s after and 3 s after glutamate application, are shown. Below, the time-course of the fluorescence change in the region of interest indicated by the red, dashed circle (upper trace) and the somatic membrane potential (lower trace) is presented. Grey bar represents time of glutamate application. No increase in Ca^2+^ is apparent upon iontophoresis of glutamate onto the axon. (d) Left, line-scan through a bouton of another cell and the corresponding Ca^2+^ transient evoked by a burst of 5 APs at 50 Hz. Right, line-scan through the same bouton during iontophoresis of glutamate. An increase in Ca^2+^ is evoked by the APs, but not by iontophoresis of glutamate.

**Figure 4:**
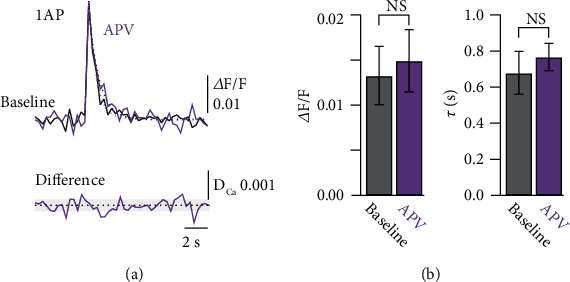
APV has no influence on presynaptic AP-evoked Ca^2+^ transients. (a) Axonal Ca^2+^ transients evoked by a single AP in a spiny stellate axon recorded in L2/3 during baseline (black) and in the presence of the NMDAR blocker APV (purple). Dashed lines represent single exponential fits to the decay of the Ca^2+^ transients. Lower trace represents the difference between the baseline and APV Ca^2+^ transients. Dashed line indicates zero and light shaded area indicates the baseline noise level (±SD). (b) Bar graph summary of the peak Ca^2+^ transient amplitudes (left) and decay time constants (right) for baseline and in the presence of APV. All data are represented as mean ± SEM.

We sought to investigate the functional consequence of preNMDAR activation and thus the potential source of Ca^2+^ required for LTD in L4 spiny stellate axons while they were activated by glutamate release from 2-AG activated astrocytes. We loaded L4 spiny stellate neurons with the Ca^2+^ indicator Oregon Green Bapta-1 (OGB-1, 200 *μ*M) and the morphological dye Alexa-594 (50 *μ*M) to trace the axon to L2/3 (Figures 2(a) and 2(b)). Frame scans of 1 min duration (3 Hz) from a stretch of axon were performed to measure the local Ca^2+^ signals before and during bath application of 2-AG. A single somatically evoked AP resulted in a stereotyped Ca^2+^ transient in the axonal compartment. Bath application of 2-AG did not cause a significant number of spontaneous, local Ca^2+^ transients in the axon as might have been hypothesized from an activation of preNMDARs (Figures 2(c) and 2(d)). We found a total of 12 spontaneous Ca^2+^ transients in 60 min of observation time (*n* = 9 cells) in the presence of 2-AG. This number was not different from spontaneous events before 2-AG application (8 events in 40 min). However, comparing the time course of the presynaptic AP-evoked Ca^2+^ transients before and after 2-AG application revealed a prolongation of the AP-evoked Ca^2+^ signal in the presence of 2-AG (Figure 2(e)). We concluded from this experiment that glutamate release from astrocytes does not activate preNMDARs in a similar manner as axonal glutamate release activates postsynaptic NMDARs, which results in clear and distinct Ca^2+^ transients in postsynaptic spines [30]. This result is consistent with observations that glutamate iontophoresis or uncaging onto presynaptic boutons does not cause a Ca^2+^ influx [13, 14]. We reconfirmed these findings by iontophoresis of glutamate (100 mM) onto dendrites and boutons of L4 spiny stellate neurons (Figure 3). We observed clear increases in Ca^2+^ in dendritic spines, but in contrast, we could not detect any Ca^2+^ elevations in the axonal compartment (*n* = 5 cells). Furthermore, single AP-evoked presynaptic Ca^2+^ signals alone were not influenced by blocking NMDA receptors. Bath-application of APV did not change the peak amplitude of the Ca^2+^ transients (Δ*F*/*F*_Baseline_ = 0.013 ± 0.003, Δ*F*/*F*_APV_ = 0.015 ± 0.003, *n* = 6, *p* = 0.22 by Student's paired *t*-test), nor its decay (*τ*_Baseline_ = 0.68 ± 0.12 s, *τ*_APV_ = 0.77 ± 0.08 s, *n* = 6, *p* = 0.55 by Student's paired *t*-test; Figure 4). Thus, preNMDARs caused no direct Ca^2+^ influx into boutons, and they also did not contribute to the normal AP-evoked presynaptic Ca^2+^ dynamics in the absence of 2-AG.

**Figure 5:**
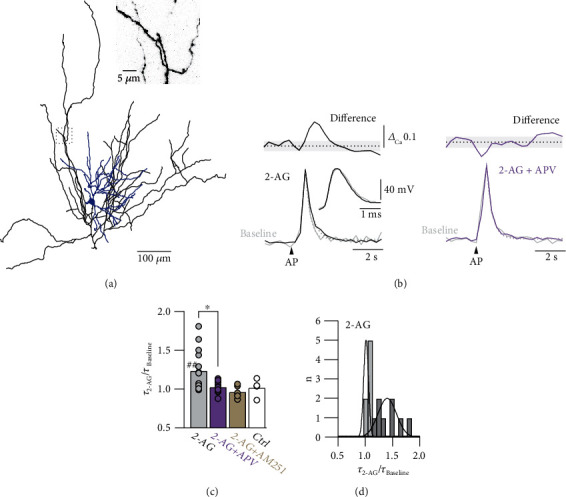
2-AG broadens AP-evoked Ca^2+^ transients in L4 axons. (a) Neurolucida reconstruction of a spiny stellate neuron. Dendrites are represented in blue and the axonal arborization in black. Inset, two-photon fluorescence image of the axon segment imaged in L2/3 indicated by the dashed box. (b) Left, averaged and normalized AP-evoked Ca^2+^ transients during baseline (grey) and after bath application of 2-AG (black). Upper trace represents the difference between the baseline and 2-AG Ca^2+^ transients. Dashed line indicates zero, and light-shaded area indicates the baseline noise level (±SD). Dashed lines represent single exponential fits to the decay of the Ca^2+^ transients. There is an apparent difference between the two transients. Inset shows somatic APs before and after bath application of 2-AG. Right, experiment in which the NMDAR-blocker APV was present in the bath. No difference between the two transients was observed in this condition. (c) Normalized decay time constant in the presence of 2-AG for different conditions. 2-AG significantly broadened the Ca^2+^ transients (*n* = 15). ^##^*p* < 0.01 for the effect of time on *τ* by paired Student's *t*-test. In contrast, no broadening was observed in the presence of APV (*n* = 11), AM251 (*n* = 5), or in control conditions without application of any drug (Ctrl, *n* = 4). APV had a significant effect on *τ* in the presence of 2-AG. ^∗^*p* < 0.05 by one-way ANOVA. All data are represented as mean ± SEM. (d) Distribution of normalized decay time constants in the presence of 2-AG. Solid black lines represent Gaussian fits to the subsets that showed either no (light grey) or significant (dark grey) broadening of the Ca^2+^ transients.

Therefore, in order to investigate the phenomenon of the specific 2-AG induced broadening of the AP-evoked Ca^2+^ signals in more detail, we measured single AP-evoked Ca^2+^ transients elicited at 0.1 Hz, corresponding to the stimulation frequency used for the LTD experiments (instead of stimulating at 0.003 Hz as in the previous imaging experiments) in spiny stellate axons before and during bath application of 2-AG (Figures 5(a) and 5(b)). We normalized and averaged the corresponding Ca^2+^ transients for comparison. We confirmed that 2-AG broadened the AP-evoked Ca^2+^ transients (Figure 5(b)). In contrast, in the presence of APV, 2-AG had no effect on the presynaptic Ca^2+^ signal (Figure 5(b)). Fitting a single exponential to the decay of the AP-evoked Ca^2+^ transients revealed a significantly slower decay in the presence of 2-AG as compared to the effect of 2-AG in the presence of APV (2-AG: normalized *τ*_2−AG_ = 1.23 ± 0.07, *n* = 15, *p* < 0.01 for the effect of time on *τ* by paired Student's *t*-test; 2-AG + APV: normalized *τ*_2−AG_ = 1.02 ± 0.02, *n* = 11, *p* = 0.26 for the effect of time on *τ* by paired Student's *t*-test; 2-AG control vs. 2-AG + APV, *p* < 0.05 by one-way ANOVA; Figure 5(c)). The distribution of the normalized decay time constants revealed that 2-AG broadened the AP-evoked Ca^2+^ transients in 53% of the axons investigated (8 out of 15 axons; average change 1.40 ± 0.08), while having no effect on the rest (7 out of 15; average change 1.03 ± 0.01; Figure 5(d)). This observation suggests that not all axons and boutons were influenced by astrocyte activation, arguing for a compartmentalized astrocytic innervation and/or for synapse-specific expression of preNMDARs. The CB1 receptor antagonist AM251 (5 *μ*M) abolished the effect of bath-application of 2-AG on the AP-evoked Ca^2+^ transients (AM251: normalized *τ*_2−AG_ = 0.97 ± 0.04, *n* = 5, *p* = 0.40 for the effect of time on *τ* by paired Student's *t*-test), excluding a nonspecific effect of 2-AG. Furthermore, repeating the experiment without any drug application had no effect on the AP-evoked Ca^2+^ transients (no drugs: normalized *τ* = 1.02 ± 0.06, *n* = 4, *p* = 0.51 for the effect of time on *τ* by paired Student's *t*-test) demonstrating long-term stability of the experimental design and ruling out any time-dependent changes in axonal Ca^2+^ buffering due to the Ca^2+^ indicator.

**Figure 6:**
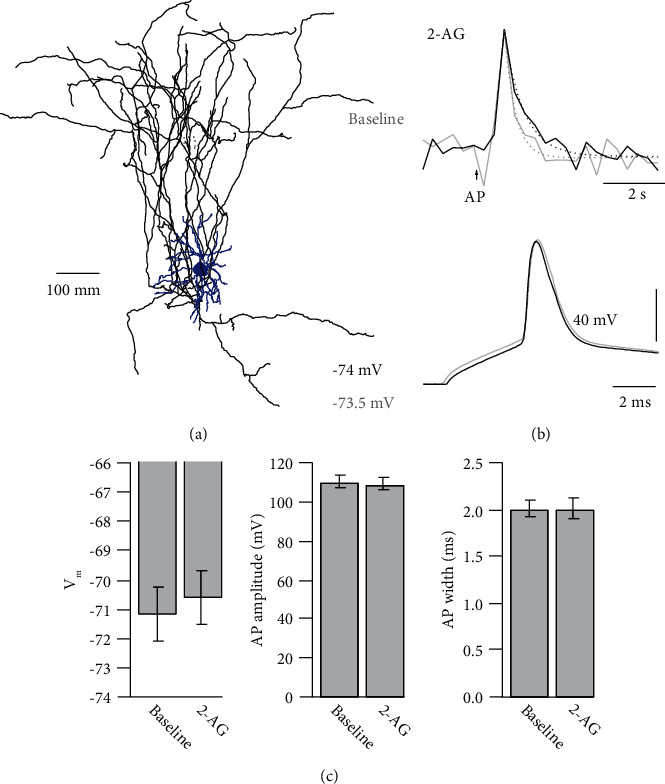
AP properties are unaffected by 2-AG. (a) Neurolucida reconstruction of a spiny stellate neuron. Dendrites are represented in blue and the axonal arborization in black. (b) Upper traces, averaged and normalized AP-evoked Ca^2+^ transients during baseline (grey) and after bath application of 2-AG (black). Dashed lines represent single exponential fits to the decay of the Ca^2+^ transients. Lower traces, corresponding somatic APs before (grey) and after (black) bath application of 2-AG. (c) Average bar graphs of resting membrane potential, AP amplitude, and AP width before and after bath application of 2-AG (*n* = 12). None of the parameters changed significantly (*p* > 0.1 by paired Student's *t*-test). All data are represented as mean ± SEM.

2-AG evoked release of glutamate might activate dendritic NMDARs on the recorded neuron, which could potentially influence somatic membrane potential, AP generation, and somatic AP properties. Previously, such somato-dendritic NMDAR activation was shown to influence presynaptic Ca^2+^ signaling in cerebellar stellate cells [12]. However, when we analyzed the somatic resting membrane potential, AP amplitude, and AP width at the soma of the imaged spiny stellate neurons, we found no influence of 2-AG on either somatic parameter (Figure 6). Therefore, we can rule out an effect of somato-dendritic NMDARs on the presynaptic Ca^2+^ dynamics. Thus, our experiments revealed an APV-sensitive presynaptic Ca^2+^ component that manifested itself in a broadening of AP-evoked Ca^2+^ transients.

**Figure 7:**
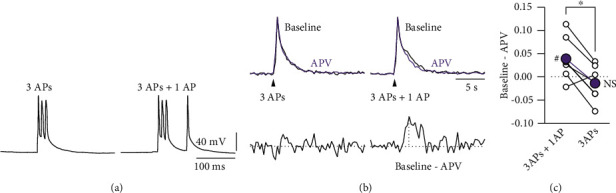
Presynaptic burst patterns evoke an APV-sensitive Ca^2+^ transient component. (a) Example of the AP burst patterns that were used to evoke presynaptic Ca^2+^ transients in L4 spiny stellate axons. Left, 3 APs at 100 Hz. Right, 3 APs at 100 Hz followed 50 ms later by a single AP. (b) Axonal Ca^2+^ transients evoked by the activity patterns shown in (a) during baseline (black) and after bath application of APV (purple). Lower traces show the difference between the corresponding transients. (c) Comparison of the peak difference between the Ca^2+^ transients before and after bath application of APV (*n* = 6). The 3AP + 1AP activity pattern showed a significant effect of APV on the evoked Ca^2+^ transients, while the Ca^2+^ transients evoked by a burst of 3 APs alone were unaffected by APV. ^#^*p* < 0.05 by paired Student's *t*-test. ^∗^*p* < 0.05 by one-way ANOVA. All data are represented as mean ± SEM.

Recently, it was shown that specific patterns of presynaptic activity alone, consisting of a burst of APs at 100 Hz or above, followed by a single AP between 50 and 200 ms later, can induce LTD. This form of LTD requires the activation of preNMDARs, but does not depend on astrocyte activation [10]. We tested whether this activity pattern also resulted in an APV-sensitive broadening of the presynaptic Ca^2+^ signal. A burst of APs at 100 Hz followed by a single AP 50 ms later evoked an axonal Ca^2+^ transient that was more rapidly decaying after wash-in of APV (Figure 7(a)). The difference between the Ca^2+^ transients under baseline conditions and in the presence of APV revealed an APV-sensitive Ca^2+^ component for this presynaptic stimulation pattern (peak difference baseline to APV: 0.04 ± 0.02, *n* = 6, *p* < 0.05 by paired Student's *t*-test; Figures 7(b) and 7(c)). Ca^2+^ transients evoked by 3 APs at 100 Hz alone showed no APV-sensitive Ca^2+^ component (−0.01 ± 0.02, *n* = 6, *p* = 0.56 by paired Student's *t*-test). Thus, the additional AP that followed 50 ms after the burst of 3 APs leads to a significant (*p* < 0.05 by one-way ANOVA) additional presynaptic Ca^2+^ influx that was abolished by APV. The requirement of the presence of this delayed 4^th^ AP suggests an interaction of the preNMDARs with a voltage-dependent mechanism initiated by the additional AP.

In summary, our experiments suggest that the activation of preNMDARs causes a slowing of the decay of AP-evoked Ca^2+^ transients, which results in an additional axonal Ca^2+^ influx that is intrinsically linked to the presence of the presynaptic AP. The source of the glutamate that activates the preNMDARs can either originate from 2-AG activated astrocytes or from glutamate spillover by a specifically timed presynaptic burst of APs.

### 3.3. preNMDARs Interact with Voltage-Dependent Ca^2+^ Channels

**Figure 8:**
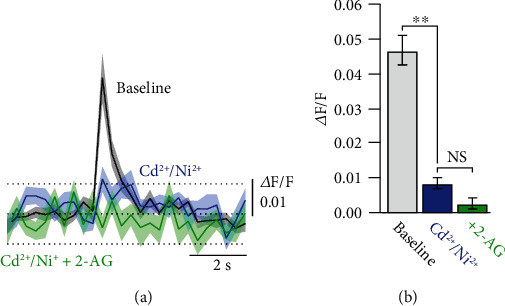
Block of VDCCs does not uncover preNMDAR-dependent Ca^2+^ transients. (a) Axonal Ca^2+^ transients evoked by a single AP in a spiny stellate axon recorded in L2/3 during baseline (black) and in the presence of the VDCC blockers Cd^2+^ and Ni^+^ (blue). Subsequent bath application of 2-AG did not result in an AP-evoked Ca^2+^ transient (green). Shaded areas represent ±SEM and dashed lines ±SD of the basal fluorescence before stimulation. (b) Bar graph summary of the peak Ca^2+^ transient amplitudes in the different conditions (*n* = 4). ^∗∗^*p* < 0.01 by Student's paired *t*-test with Bonferroni correction for multiple comparisons. All data are represented as mean ± SEM.

The broadening of the AP-evoked presynaptic Ca^2+^ transient could directly be due to the preNMDARs, which could contribute to Ca^2+^ influx during the AP-evoked axonal membrane depolarization and the subsequent relief of the Mg^2+^ block. In this scenario, preNMDARs would function as classical coincidence detectors, similar to what has been suggested for postsynaptic NMDARs. However, it has been shown that preNMDARs during early development might contain the GluN3A subunit, which renders them largely insensitive to Mg^2+^ block and with low permeability for Ca^2+^ [18]. An alternative function of the preNMDARs might be to contribute to the axonal membrane depolarization by Na^+^ influx or to exert a metabotropic effect during their activation. Both mechanisms could activate voltage-dependent Ca^2+^ channels (VDCCs) beyond the membrane depolarization caused by the axonal AP and contribute to an additional Ca^2+^ influx. To distinguish between a direct Ca^2+^ influx through preNMDARs and an interaction with VDCCs, we performed axonal Ca^2+^ imaging as described above and blocked VDCCs (Figure 8). Somatic APs evoked a consistent axonal Ca^2+^ transient which was abolished in the presence of the VDCC-blockers Cd^2+^ (100 *μ*M) and Ni^+^ (50 *μ*M) even though the somatic AP waveform was unchanged (Δ*F*/*F*_Baseline_ = 0.047 ± 0.004, Δ*F*/*F*_Cd,Ni_ = 0.008 ± 0.001, *n* = 4, *p* < 0.01 by Student's paired *t*-test with Bonferroni correction for multiple comparisons). Subsequent continuation of somatic AP stimulation during bath application of 2-AG, but still in the presence of Cd^2+^/Ni^+^ did not uncover an AP-evoked Ca^2+^ transient through preNMDARs (Δ*F*/*F*_2−AG_ = 0.002 ± 0.001, *p* = 0.32 by Student's paired *t*-test with Bonferroni correction for multiple comparisons). This observation suggests that axonal membrane depolarization is not required for unblocking preNMDARs from a putative Mg^2+^ block to render them permeable for Ca^2+^. Contrary, we conclude that functional VDCCs are required so that preNMDARs can interact with them to prolong the axonal Ca^2+^ influx.

## 4. Discussion

NMDARs are essential ionotropic glutamate receptors for synaptic transmission, information processing, and synaptic plasticity. While classically thought to be located mainly postsynaptically, there is growing anatomical and physiological evidence that NMDARs also have important functions at presynaptic sites [2, 31–38]. We investigated the function of preNMDARs in juvenile L4-to-L2/3 glutamatergic connections in the somatosensory cortex. First, we showed that activation of astrocytes with the endocannabinoid 2-AG resulted in a form of presynaptic LTD (eCB-LTD) that depended on astrocyte Ca^2+^ signaling and the activation of preNMDARs, thereby showing an overlapping mechanism of induction with t-LTD [8]. Recording presynaptic Ca^2+^ dynamics during the induction of eCB-LTD allowed us to investigate the functional consequences of preNMDAR activation. In line with other studies, we found no evidence for a direct Ca^2+^ influx through preNMDARs during eCB-LTD induction or glutamate iontophoresis [13, 14]. Instead, our results suggest that the activation of preNMDARs leads to a prolonged activity of VDCCs resulting in an additional AP-evoked Ca^2+^ influx through these channels. Thus, we conclude that the action of preNMDARs has an indirect influence on presynaptic Ca^2+^ transients by interacting with VDCCs. These findings can reconcile some of the controversial results regarding preNMDARs and are consistent with the electrophysiological evidence for their influence in t-LTD.

### 4.1. Signaling Cascade for the Induction of t-LTD at Developing Cortical Synapses

The chemically induced eCB-LTD presented here shares the same signaling cascade as found in t-LTD. In t-LTD, the eCB 2-AG is synthetized by postsynaptic AP firing followed by presynaptic glutamate release. The postsynaptic backpropagating AP evokes an increase in postsynaptic Ca^2+^ through VDCCs, which is thought to prime phospholipase C (PLC), which is subsequently activated by the presynaptic release of glutamate binding to the metabotropic glutamate receptor type 5 (mGluR5) [6, 7]. In eCB-LTD, this postsynaptic signaling cascade is circumvented. However, the pathway downstream from eCB production is the same: in both cases, the activation of astrocytes by 2-AG resulting in an increase in astrocyte Ca^2+^ activity is necessary. Furthermore, preNMDARs are required for both t-LTD and eCB-LTD. preNMDARs are expressed in a target-cell-specific way only at a subset of synapses. This suggests that preNMDAR-mediated plasticity is limited to specific neuronal connections [19, 39–41]. Accordingly, we only found a 2-AG induced broadening of the Ca^2+^ transients in a subset of the investigated axonal boutons.

The presynaptic AP is an essential component for eCB-LTD induction, since without the interaction of the AP with preNMDAR activation, there is no change in the presynaptic Ca^2+^ signal. This is in line with our earlier observation that when LTD is induced by direct electrical stimulation of astrocytes (thereby circumventing the necessity of endocannabinoid signaling), this LTD still requires presynaptic AP firing during the astrocyte activation [8]. This observation can now be explained, since only the interaction of the preNMDAR with VDCCs, activated by the axonal AP, changes the presynaptic Ca^2+^ dynamics. This in turn presumably leads to calcineurin modulation and LTD. Interestingly, very similar results have been obtained by others. In the first study showing involvement of eCBs and preNMDARs in t-LTD, it was already shown that eCB application only led to LTD if it was paired with presynaptic activity [4]. Furthermore, eCB-mediated LTD at inhibitory synapses in the hippocampus, which also requires calcineurin activity, shares the requirement for AP firing in the presynaptic neuron for its induction [27].

A similar interaction of a presynaptic ionotropic glutamate receptor being activated by astrocytes and influencing synaptic release has recently been demonstrated [42]. In this case, axonal AMPARs were shown to be activated by astrocytes and contributed to axonal depolarization, broadening the axonal AP and thus influencing the Ca^2+^ dynamics at presynaptic sites. Furthermore, several studies have shown that somatic depolarization can lead to an additional axonal depolarization that gives rise to graded, analog release of transmitter [43, 44]. Importantly, we did not find an influence of 2-AG on the somatic membrane potential nor on AP properties, thereby ruling out such an influence on the axonal Ca^2+^ signals in our experiments.

It should be noted that an intriguing interaction of postsynaptic NMDAR activation with presynaptic Ca^2+^ dynamics has also been described [45]. At hippocampal CA3-CA1 synapses, the efflux of potassium through postsynaptic NMDARs provides a retrograde signal to the presynaptic bouton, which can boost the presynaptic AP-evoked Ca^2+^ transient and increase neurotransmitter release. However, we deem it unlikely that a similar mechanism involving postsynaptic NMDAR activation can explain our observations. First, experiments with MK801 in the pre- or postsynaptic neuron show that both t-LTD [5, 8] and eCB-LTD (this study) require presynaptic, not postsynaptic, NMDAR activation. These results are supported by the finding that t-LTD at L4-L2/3 synapses in developing visual cortex is disrupted by cell-type-specific removal of NMDARs specifically from presynaptic L4 neurons [40]. Therefore, evidence for involvement of pre- rather than postsynaptic NMDARs in L4-L2/3 LTD is quite strong. Furthermore, when potassium-mediated retrograde signaling at CA3-CA1 axons was studied a Ca^2+^ transient broadening mediated by postsynaptic NMDARs was only observed with repetitive AP firing in the absence of extracellular Mg^2+^ [45]. In contrast, in our experiments, the 2-AG-mediated broadening of Ca^2+^ transients in L4 boutons occurred with single AP firing in the presence of 1 mM extracellular Mg^2+^. Under our experimental conditions, the potassium efflux through postsynaptic NMDARs is likely minimal due to Mg^2+^ block of these receptors. Finally, we observed that not all L4 boutons were showing a 2-AG induced broadening of the presynaptic Ca^2+^ transient. This is similar to what was observed in excitatory boutons in L5 of developing neocortex [35]. It indicates that not all L4 boutons contain preNMDARs. If postsynaptic NMDARs would be responsible for the presynaptic Ca^2+^ transient broadening such a lack of effect in some boutons is harder to explain, since postsynaptic NMDARs are ubiquitously expressed at most glutamatergic synapses [46–49].

It was recently shown that a presynaptic burst of APs followed by a single AP between 50–200 ms later can also trigger LTD (termed pattern dependent LTD, p-LTD) [10]. The presynaptic burst of APs is probably sufficient to cause spillover of presynaptically released glutamate onto preNMDARs, supported by the findings that p-LTD no longer requires astrocyte activation, but still depends on preNMDARs. Presumably, the single AP occurring with a delay comes at the time when the presynaptically released glutamate from the preceding burst has activated preNMDARs. Consistently, when we performed presynaptic Ca^2+^ imaging, we were able to show that the p-LTD presynaptic activity pattern evoked an APV-sensitive Ca^2+^ component, whereas a burst of 3 APs alone did not. Thus, preNMDARs can differentially be activated depending on the pattern of presynaptic activity and only contribute to an additional Ca^2+^ influx under certain conditions.

An interesting question in this context is which type of VDCC is modulated by the preNMDARs? Previous experiments suggest that neither L-type nor R-and T-type VDCCs are required, because t-LTD can be induced in the presence of blockers of these channels using a burst of 3 postsynaptic APs followed by a single presynaptic AP at -10 ms [6]. Single postprepairings are sensitive to these blockers suggesting a role of these VDCCs in the postsynaptic signaling cascade [6, 7]. Thus, N- and P/Q-type VDCCs might interact with the preNMDARs.

Our data is in line with the idea that preNMDAR-mediated depolarization of the terminal carried by axonal Na^+^ influx through the receptor plays a role in the interaction of preNMDARs with VDCCs. A similar conclusion on the importance of NMDAR-mediated Na^+^ influx was reached for the effect of preNMDARs on spontaneous synaptic release [15]. This ionotropic effect of preNMDARs is further supported by the finding that presynaptically applied MK801, which acts as an open channels blocker and preventing ion flow through the NMDAR, is effective in blocking t-LTD [5, 50]. Similarly, presynaptic MK801 application also affects direct modulation of release through preNMDARs [19]. This efficacy of MK801 in blocking preNMDAR effects makes a metabotropic role as has been suggested recently for hippocampal LTD for these receptors [16, 51] unlikely since lack of MK801 block is seen as a hallmark for metabotropic NMDAR function (but see below for an alternative interpretation).

### 4.2. Conflicting Data on the Existence of preNMDARs

Several studies to date have sought for functional evidence of preNMDARs in neocortex and have come to the conclusion that these receptors do not exist [13, 14]. Our current results, together with earlier findings, offer an alternative explanation for this apparent controversy. Our study suggests that the function of preNMDARs differs from the classical coincidence detector role as described for postsynaptic NMDARs. Postsynaptic NMDAR activation requires the relieve of the Mg^2+^ block by a backpropagating AP to supralinearly enhance postsynaptic Ca^2+^ influx [30]. In contrast, we find little evidence for a direct preNMDAR-mediated Ca^2+^ signal. This is in line with several findings about the subunit composition of preNMDARs at developing synapses in the neocortex. In the developing visual cortex, preNMDARs contain the NR3A subunit rendering these NMDARs insensitive to Mg^2+^ and little Ca^2+^ permeable [18, 40]. Both properties agree with our findings that without a presynaptic AP, there is no substantial Ca^2+^ influx through preNMDARs. The lack of a presynaptic Ca^2+^ signal by iontophoresing glutamate onto presynaptic boutons or by uncaging of MNI-glutamate was interpreted by others as a lack of preNMDARs [13, 14]. However, our findings suggest that the effect of the preNMDARs on axonal Ca^2+^ signaling is rather subtle and becomes only apparent in the presence of specific patterns of presynaptic APs, thereby explaining the apparent lack of preNMDAR activity in other studies. Similar results were obtained by others when performing Ca^2+^ imaging experiments at glutamatergic synapses onto cortical interneurons: only prolonged axonal activation with sustained bursts of APs clearly uncovered an APV-sensitive component in the Ca^2+^ transient [19]. At other central synapses, direct Ca^2+^ influx has been observed through preNMDARs suggesting that there is a synapse-specific differential subunit composition of preNMDARs [20].

Importantly, the recent conclusion that t-LTD requires post- rather than presynaptic NMDARs was not just based on negative Ca^2+^ imaging data but also on the absence of L4-L2/3 t-LTD in a transgenic mouse in which L2/3 NMDARs were selectively disrupted [14]. Although we cannot explain this apparent discrepancy, it should be noted that another study using a transgenic mouse in which L4 NMDARs were instead selectively disrupted also showed a disruption of L4-L2/3 t-LTD [40], thereby illustrating the potential developmental, species-specific, and brain-region specific differences, which are observed in these experiments.

Finally, pharmacological evidence presented recently by Carter and Jahr [14] suggests that the mechanism of action of NMDARs involved in t-LTD is metabotropic. This conclusion was based on the inability of extracellularly applied MK-801 to block t-LTD, as well as on a lack of block by the glycine-site antagonists 7-CK and 5,7-DCK. The finding that extracellular MK-801 does not block t-LTD is in direct contradiction with studies showing effective t-LTD block by intracellularly applied MK-801 [5, 50]. In this respect, it is important to note again that the pharmacological profile of preNMDARs might differ from that of “classical” postsynaptic (NR1 and NR2 containing) NMDARs. Incorporation of the NR3A subunit (presumably in triheteromeric NR1-NR2B-NR3A receptors [18]) might alter receptor pharmacology (e.g., of MK801), possibly explaining such contradictory results [52]. However, our findings cannot distinguish between an ionotropic or a metabotropic role for preNMDARs [53]. If preNMDARs have a metabotropic function, they could exert their effect by a direct interaction with VDCCs to facilitate Ca^2+^ influx or by an inactivation of presynaptic K^+^ channels, both of which could broaden the AP locally and thus enhance presynaptic Ca^2+^ influx [54], which would be the required signal for calcineurin activation.

## 5. Conclusion

In summary, we show evidence for the existence of functional preNMDARs in spiny stellate axons at L4-L2/3 synapses in the developing rat barrel cortex. Their function is to sense glutamate either released from astrocytes or from spill-over by enhanced presynaptic activity and then to modulate the local axonal Ca^2+^ influx. This modulation could be either by contributing to the local membrane depolarization or by a metabotropic action affecting VDCCs or other presynaptic ionic conductances. Either mechanism would affect subsequent axonal APs by modifying AP-induced Ca^2+^ dynamics, thereby leading to the induction of LTD. The elucidation of the mode of action of preNMDARs is one of the remaining missing pieces to understand the signaling cascade of t-LTD at developing cortical synapses.

## Data Availability

Data is available on request from the corresponding author.
